# Covid conspiracies: misleading evidence can be more damaging than no evidence at all

**DOI:** 10.1017/S0033291720002184

**Published:** 2022-02

**Authors:** Sally McManus, Joanna D'Ardenne, Simon Wessely

**Affiliations:** 1 School of Health Sciences, City, University of London, London, UK; 2 National Centre for Social Research. Joanna d'Ardenne Questionnaire Development and Testing Hub, National Centre for Social Research, London, UK; 3 Department of Psychological Medicine, King's College London, London, UK

## Letter to the editor

We read with interest the article by Freeman and colleagues ‘Coronavirus Conspiracy Beliefs, Mistrust, and Compliance with Government Guidelines in England’ (2020).

It aimed to estimate the prevalence of conspiracy thinking about the pandemic and test associations with reduced adherence to government guidelines. Appropriately, the authors focused on the latter aim in their text. However, the tables present information on prevalence of specific types of conspiracy thinking and this featured in the press release, picked up globally with headlines like ‘One fifth of English people blamed Jews or Muslims for COVID-19’ (Newsweek, 2020).

The paper actually suggests that a fifth of adults in England blamed Jews for COVID-19 *and* a fifth blamed Muslims. Also that a fifth held aliens responsible, a fifth thought Bill Gates started it, a fifth claimed it was Big Pharma, a fifth said 5G caused it, and a quarter agreed the virus was manufactured by the World Health Organisation and United Nations to take global control. And that's before we get to China's role. Further, more than a fifth agreed ‘The virus is a smokescreen for a global conspiracy that swapped the real world with a simulation.’

Several commentators have observed that this doesn't quite pass the smell test (e.g. Pollard, 2020). The results are all the more surprising given their divergence from other studies. One poll conducted a few weeks earlier found 7% of adults thought 5G played a role in the pandemic (Opinium, 2020).

So what's going on? A lifetime of conducting and reading surveys suggests the following maxim − the more dramatic and headline catching the finding, the greater the need to scrutinise the methodology. And this paper seems to be no exception to this rule of thumb.

When framing response options in attitudinal research, a balance of agree and disagree response options is standard practice, e.g. strongly and slightly disagree options, one in the middle, and two in agreement. Some respondents avoid the ‘extreme’ responses either end of a scale. But here, there was just one option for ‘do not agree’, and four for agreement (agree a little, agree moderately, agree a lot, agree completely). Acquiescence response bias is the well-established tendency for survey respondents to agree with statements, regardless of their content (Hibbing, Cawvey, Deol, Bloeser, & Mondak, 2019).

Any prevalence estimate is only as good as the sample it's based on (Pierce et al., 2020). The authors fully acknowledge the limitations of the non-probability sample. Advertised invitations to participate introduced the study as about different explanations for COVID-19, likely therefore to appeal to those who have different explanations. Furthermore, 20% of the sample were children (no lower age of participation is given), and the children taking part were particularly likely to endorse conspiracy theory beliefs.

If the results are inaccurate, this matters. Of course it is embarrassing to have headlines around the world claiming that a fifth of people in England believe ‘Jews have created the virus to collapse the economy for financial gain.’ But it's dangerous too. ‘Findings’ that indicate fringe beliefs are more widely held than they actually are can serve to normalise those beliefs. And can stoke fear among the groups being blamed.

Misleading evidence can be more damaging than no evidence at all.
